# Investigation of antiviral substances in Covid 19 by Molecular Docking: In Silico Study

**DOI:** 10.4314/ahs.v23i1.4

**Published:** 2023-03

**Authors:** Erkan Oner, Ilter Demirhan, Meral Miraloglu, Serap Yalin, Ergul Belge Kurutas

**Affiliations:** 1 Department of Biochemistry, Faculty of Pharmacy, Mersin University, Mersin, 33090, Türkey; 2 Vocational School of Health Services, Department of Electronics-Automation Biomedical Device Tecnology Program, Harran University, Sanlıurfa, 63090, Türkey; 3 Department of Medical Microbiology, Cukurova University, Medical Faculty, Adana, Türkey; 4 Department of Biochemistry, Faculty of Pharmacy, Mersin University, Mersin, 33090, Türkey; 5 Department of Medical Biochemistry, Faculty of Medicine, Sutcu Imam Univestiy, Kahramanmaras, 46090, Türkey

**Keywords:** Sars-CoV-2 Main Protease, Antiviral Drugs, Molecular Docking

## Abstract

**Aims:**

This paper aimed to investigate the antiviral drugs against Sars-Cov-2 main protease (MPro) using in silico methods.

**Material and Method:**

A search was made for antiviral drugs in the PubChem database and antiviral drugs such as Bictegravir, Emtricitabine, Entecavir, Lamivudine, Tenofovir, Favipiravir, Hydroxychloroquine, Lopinavir, Oseltamavir, Remdevisir, Ribavirin, Ritonavir were included in our study. The protein structure of Sars-Cov-2 Mpro (PDB ID: 6LU7) was taken from the Protein Data Bank (www.rcsb. Org) system and included in our study. Molecular docking was performed using AutoDock/Vina, a computational docking program. Protein-ligand interactions were performed with the AutoDock Vina program. 3D visualizations were made with the Discovery Studio 2020 program. N3 inhibitor method was used for our validation.

**Results:**

In the present study, bictegravir, remdevisir and lopinavir compounds in the Sars-Cov-2 Mpro structure showed higher binding affinity compared to the antiviral compounds N3 inhibitor, according to our molecular insertion results. However, the favipiravir, emtricitabine and lamuvidune compounds were detected very low binding affinity. Other antiviral compounds were found close binding affinity with the N3 inhibitor.

**Conclusion:**

Bictegravir, remdevisir and lopinavir drugs showed very good results compared to the N3 inhibitor. Therefore, they could be inhibitory in the Sars Cov-2 Mpro target.

## Introduction

Severe acute respiratory syndrome coronavirus 2 (Sars-Cov-2) infection has become an epidemic and has affected the whole world. This epidemic has been named as Coronavirus Disease -2019 (COVID-19) by the World Health Organization (WHO). The COVID-19 pandemic has spread very rapidly all over the world and has caused many people to die 1. COVID-19 disease is caused by a new coronavirus and is called a respiratory disease. First seen in China, this disease is highly contagious and its main clinical symptoms include fever, dry cough, fatigue, muscle pain, and shortness of breath. Since the first reported case of COVID-19 in Wuhan, China, at the end of 2019, it seems to have affected all the countries of the World 2. Families, societies and countries have been adversely affected by COVID-19. The concepts of health, freedom, economy, medicinal plants have gained great importance.

**Figure 1:**
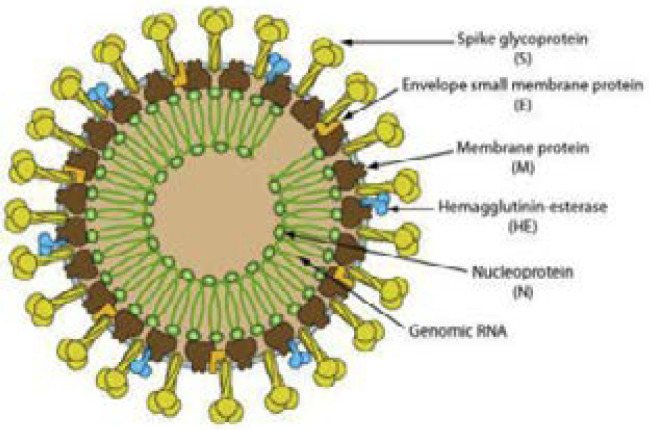
Covid 19 Structure 8.

COVID-19 can disrupt the healthcare systems of many countries. While the number of confirmed COVID-19 cases on 22 January was 580, these figures have now risen to 18.3 million worldwide (August 5) 3. Transmission can occur between people through oral and nasal droplets and through contact with contaminated surfaces 4 For example, from a cough droplet in an aerosol can spread 4 to 5 m away, while sneezing can spread droplets 8 m away 5. Coronaviruses are positive polarity, enveloped, single-stranded RNA viruses with rod-like projections on the surface. According to their genomic structures, coronaviruses are divided into alpha (α), beta (β), gamma (γ) and delta (δ) subgroups. Sars-Cov, Middle East Respiratory Syndrome Coronavirus (MERS-CoV) and Sars-Cov-2 viruses are in the subgroup of β coronaviruses 6. Sars-Cov-2 is a virus that can synthesize at least 14 genetic code sequences (ORF, open-reading frames) with a 30 kb genome. It has 4 structural proteins: Spike (S), Envelope (E), Membrane (M) and Nucleocapsid (N). These proteins are used as targets in vaccine and drug development in the treatment of COVID-19 7. (Figure 1).

Drug repositioning, reuse, reprofiling, or reassignment is the evaluation of existing drugs for new therapeutic purposes^9^. A candidate drug (investigational or approved) for reuse efforts should at least be known based on successful Phase I or Phase II clinical trials. It has a safety and toxicity profile 10. Considering the whole process, it is estimated that the costs to market a tailored drug are ten times lower and the time to market is shortened by about half, compared to a new drug 11. While developing a new drug requires lengthy processes such as discovery and research, preclinical studies, clinical trials, and treatment approval, reuse studies of previously discovered drugs have many advantages over a new drug development study: less time for development, financial investment, and lower cost. there is a risk of failure 12.

Viruses that have emerged or will re-emerge pose major public health concerns globally 11 For several pathogenic viruses, there are significant efforts focused on vaccine development 13, 14. However, those infected during pandemics urgently need broad treatment. Different drug stores are urgently needed for the COVID-19 outbreak. Therefore, drug reuse may be one of the best strategies to deal with this pandemic 15,16. Computational and experimental approaches can be used alone or in combination to gain a more holistic view and increase the chances of success in drug reuse.

There is not enough evidence yet for the use of favipiravir, hydroxychloroquine, Lopinavir, in the treatment of COVID-19 infection due to the weak power of evidence (not blinding, lack of control group, use of many other drugs, unsimilar baseline patient criteria, etc.).

Our aim in this study, which was done for the first time, is to find the binding activities of these drugs by directing new drugs to the target with computer-aided drug design in addition to the antiviral drugs used in COVID-19.

## Material and Method

### Molecular Modeling Studies

#### Ligand System

Antiviral drugs were taken from PubChem (https://pubchem.ncbi.nlm.nih.gov) database in sdf format. Converted from Open Babel GUI program to pdb format (Table 1).

**Table 1:** Antiviral drugs present in for docking studies

Sr. No.	Antiviral Drugs	Formula	Compound ID
**1**	Bictegravir	C_21_H_18_F_3_N_3_O_5_	90311989
**2**	Emtricitabine	C_8_H_10_FN_3_O_3_S	60877
**3**	Entecavir	C_12_H_15_N_5_O_3_	135398508
**4**	Lamivudine	C_8_H_11_N_3_O_3_S	60825
**5**	Tenofovir	C_9_H_14_N_5_O_4_P	464205
**6**	Oseltamavir	C_16_H_28_N_2_O_4_	65028
**7**	Ribavirin	C_8_H_12_N_4_O_5_	37542
COVID-19 Antiviral Drugs			
**8**	Favipiravir	C_5_H_4_FN_3_O_2_	492405
**9**	Hydroxychloroquine	C_18_H_26_CIN_3_	2719
**10**	Lopinavir	C_37_H_48_N_4_O_5_	92727
**11**	Remdevisir	C_27_H_35_N_6_O_8_P	121304016
**12**	Ritonavir	C_37_H_48_N_6_O_5_S_2_	39622

#### Protein system

Crystal structures of protein structures were obtained from Protein Data Bank (www.rcsb. Org). All polar hydrogens have been added with the Discovery Studio 2020^15^ modeling package to reduce the tension of the crystal structure and make the proteins available for use in the Autodock simulation program. The structure obtained has been minimized in vacuum environment; during minimization, the heavy atoms are fixed at the initial crystal coordinates; the hydrogens are released to allow them to move. Autodock tools graphical user interface program was used to prepare proteins and ligands. Gasteiger charges were calculated and non-polar hydrogens joined with carbon atoms. For the macromolecules, the generated pdbqt files are saved (Table 2). Protein structure of 6LU7 or N3 inhibitor (N-[(5-methylisoxazoL-3-YL) Carbonyl] Alanyl-L-Valyl-N∼1∼-((1R,2Z)-4-(Benzyloxy))-4-Oxo-1-{[(3R)-2-Oxopyrrolidin-3-YL] Methyl} But-2-Enyl)-L-leucinamide) is a complex structure.

**Table 2:** Targeted receptor protein (PDB ID:6LU7) associated with COVID19 along with structure

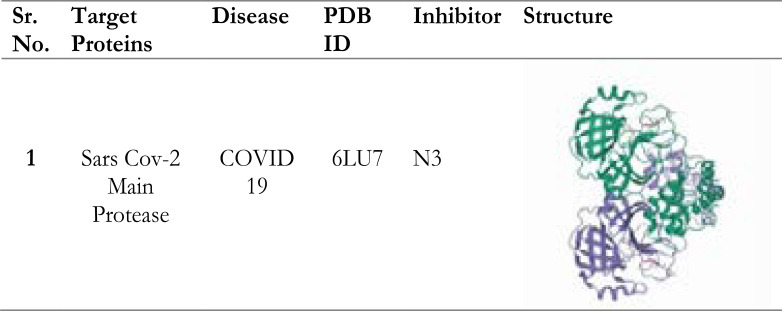

### Molecular Modeling

Autodock 4.2.6 was used. AutoDock tools program was used to create modeling data entry files. In all models, a cube divided into squares with 80x80x80 point dimensions in x, y, z directions were created. A length of 0.375 Å (approximately one quarter of the length of the carbon-carbon covalent bond) and a distance-dependent function of the dielectric constant were used to calculate the energy of the mappings. 10 processes were carried out using Lamarckian genetic algorithm logic. Randomly placed fragments with an initial population of 50 were used with a maximum energy of 2.5 x 106 and a maximum of 2.7 x 104 occurrences. A mutation rate of 0.02 and a genetic change rate of 0.8 were chosen. Results differing by less than 0.5 Å in the root mean square deviation (RMSD) were pooled together and the results of the optimal free energy of binding were chosen as the final complex structures. Using Autodock Vina 1.1.2 16. and Discovery Studio 2020 programs, ligand-protein interactions were investigated 17.

### Validation Method

N3 inhibitor from Sars Cov-2 inhibitor protein (belonging to PDB ID:6LU7) was extracted with AutoDock 4. Docking was performed without adding N3 inhibitor, which is Sars Cov-2 Mpro ligand. Also, the mean square of difference (RMSD) value in PyMOL is checked to see the validation of our study. If the RMSD value is less than 2.0 Å, it indicates that the method is valid. This can manage to reduce the cost 18

## Results

**Figure 2:**
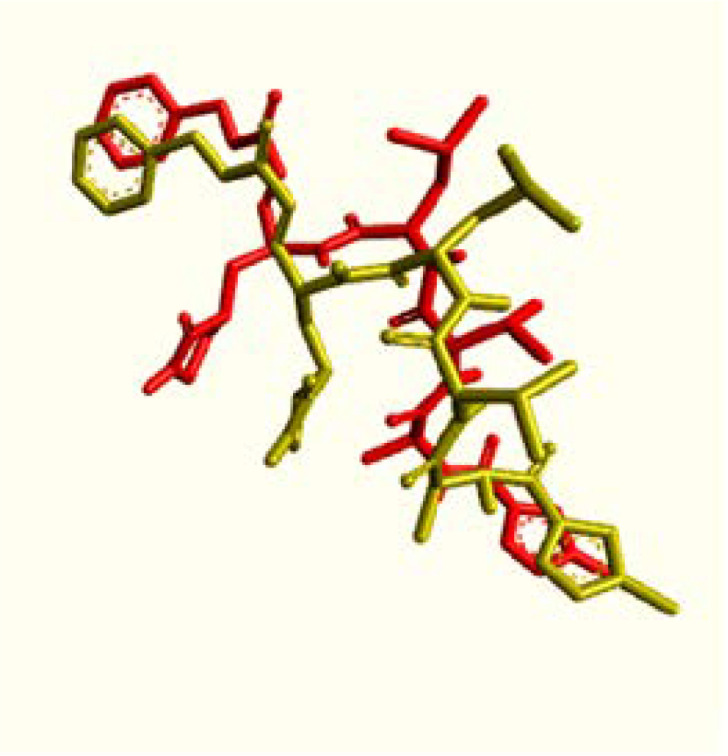
Status of N3 inhibitor with Sars-Cov-2 MPro inhibitor before reconfirmation (red), status of N3 inhibitor after insertion (yellow)

Revalidation with N3 inhibitor was performed to reveal the strength of binding affinity. The result of our validation is shown in Figure 2. The ligand had an RMSD value of 1.61 Å and the binding energy was -6.9 kcal/mol. This molecular docking is valid due to the RMSD value 2.0 Å below the method used.

Docking results of selected antiviral drugs with target protein Sars-Cov-2 Mpro showed that selected antiviral drugs had a good binding affinity and better binding modes than that standard drugs against selected target receptors. The results of docking score of selected antiviral drugs with target proteins is presented in the Table 3.

**Table 3:** Docking score antiviral drugs against target protein receptors (Docking score was expressed in terms of kcal/mol)

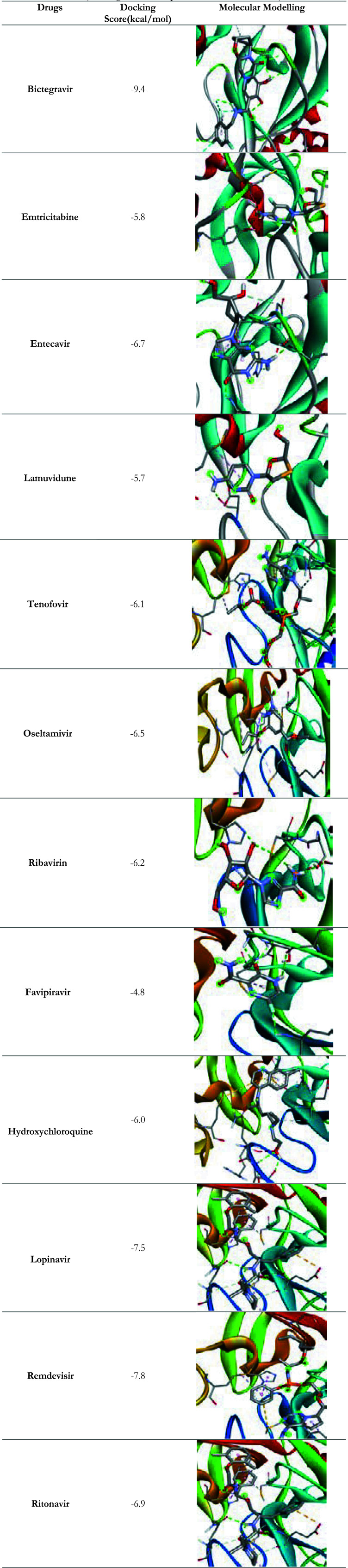

Sars-Cov-2 Mpro (PDB ID: 6LU7) binding structures of antiviral drugs are given in Table 4.

**Table 4:** Discovery Studio structure showing interactions between anti viral drugs and target proteins (PDB ID: 6LU7)

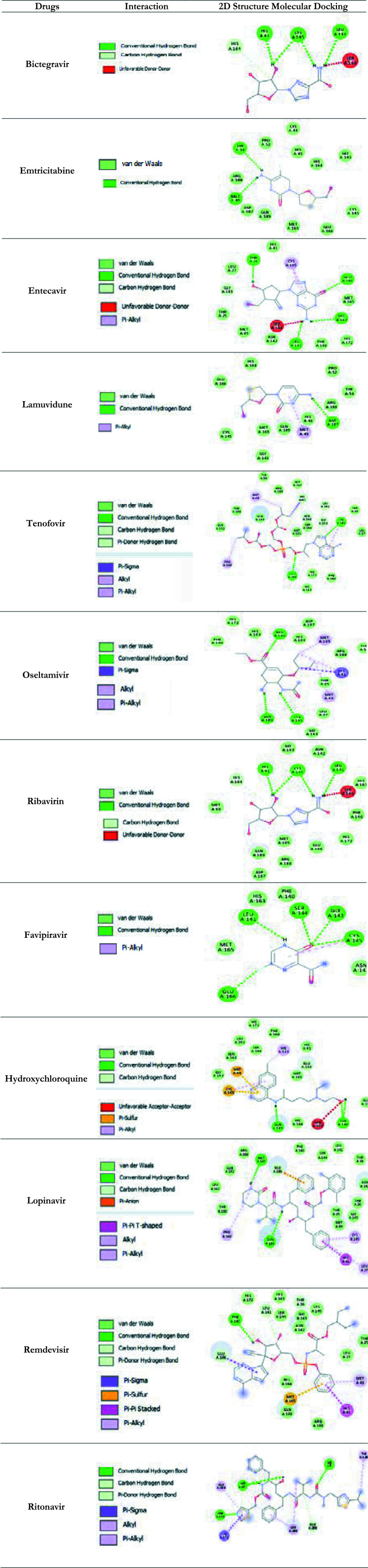

The H bond and hydrophobic interaction between the Sars Cov-2 Mpro receptor and antiviral compounds appear in Table 5.

**Table 5:** H-bond and hydrophobic interaction between high binding score compounds and receptors

Drugs	H-Bond Interactions	Hydrophobic Interactions
**Bictegravir**	HIS41, LEU141, CYS145, HIS164	-
**Emtricitabine**	MET49, TYR54	HIS41, CYS44, PRO52, GLY143, CYS145, HIS164, MET165, GLU166, ASP187, ARG188, GLN189
**Entecavir**	THR26, LEU141, HIS163, GLY144, GLU166	THR25, LEU27, HIS41, MET49, PHE140, ASN142, MET165, HIS170
**Lamuvidune**	ASP187	HIS41, PRO52, TYR54, GLY143, CYS145, HIS164, MET165, GLU166, ARG188, GLN189
**Tenofovir**	HIS41, CYS145, LEU141, ASN142, GLY143, GLU166	THR26, LEU27, TYR54, PHE140, SER144, HIS163, MET165, HIS172, ASP187, ARG188, GLN189, THR190, GLN192
**Oseltamivir**	CYS145, GLU166, GLN189	THR25, LEU27, TYR54, PHE140, GLY143, HIS163, HIS164, HIS172, ASP187, ARG188
**Ribavirin**	HIS41, LEU141, CYS145, HIS164	MET49, PHE140, ASN142, GLY143, GLU166, MET165, HIS172, ASP187, ARG188, GLN189
**Favipiravir**	LEU141, GLY143, SER144, CYS145, GLU166	PHE140, ASN142, HIS163, MET165
**Hydroxychloroquine**	GLU166, GLN189, THR190	HIS41, PHE140, LEU141, ASN142, GLY143, SER144, HIS164, MET165, HIS172
**Lopinavir**	MET165, GLU166, GLN189	TYR24, THR25, THR26, MET49, PHE140, LEU141, ASN142, GLY143, SER144, LEU167, ARG188, THR190, GLN192
**Remdevisir**	THR26, PHE140, LEU141, GLU166	THR25, LEU27, ASN142, GLY143, SER144, CYS145, HIS163, HIS164, HIS172, ARG188, GLN189
**Ritonavir**	LYS5, ASN133, ASP197, GLU288	-

According to the results of the molecular docking study, the inhibitory contents and RMSD values of antiviral drugs are shown in Table 6.

**Table 6:** Autodock 4.2-RMSD Inhibition Constant of Molecular Docking Study

Sr. No.	Antiviral Drugs	Inhibition Constant, Ki(mM)	RMSD (Å)
**1**	Bictegravir	33.41	0.31
**2**	Emtricitabine	201.47	0.41
**3**	Entecavir	65.24	0.78
**4**	Lamivudine	282.04	1.98
**5**	Tenofovir	98.67	1.12
**6**	Oseltamavir	133.02	0.84
**7**	Ribavirin	168.45	1.34
COVID-19 Antiviral Drugs			
**8**	Favipiravir	231.59	0.66
**9**	Hydroxychloroquine	221.58	0.95
**10**	Lopinavir	141.70	0.99
**11**	Remdevisir	74.32	0.44
**12**	Ritonavir	53.32	0.71

## Discussion

It is seen that a wide variety of strategies are used in the treatment of COVID-19 disease, which has turned into a pandemic due to the very recent emergence of Sars-Cov-2 infection, which has no proven treatment. Many of these emerging strategies are based on the reuse of existing drugs that are effective in different indications. Chloroquine analogue (i.e., hydroxychloroquine) inhibit endosomal acidification (increasing pH) necessary for virus-cell fusion, and are in vitro non-invasive against many viruses such as HIV, Dengue, hepatitis C, Chikungunya, Influenza, Ebola, SARS, MERS and COVID-19 agents. It has been found to have a specific antiviral effect. It has been shown in studies with Vero cells that its effect is both at cell entry and after entry. It is thought that having an immunomodulatory effect at the same time increases its antiviral effect. It is a drug that spreads throughout the body, including the lungs 19.

Favipiravir, a selective RNA-dependent RNA polymerase inhibitor, is an antiviral used in the treatment of influenza in some Asian countries. Apart from influenza, it also has an inhibitory effect against many viruses such as arena-, bunya-, flavi- and filoviruses, and hemorrhagic fever viruses such as Ebola 20. Therefore, it was thought to be effective during the Covid-19 pandemic and was offered for use in cases with severe clinical findings in our country.

Lopinavir/ritonavir (LPV/r) is a protease inhibitor used in the treatment of HIV/AIDS. The protease enzyme is a key enzyme for the polyprotein formation of the coronavirus. Although conflicting results have been obtained in the treatment of SARS, it has been one of the antiviral drugs that hope in the treatment of Covid-19, due to its strong effect in vitro and in vivo, especially with interferon-beta (IFNb) for MERS-CoV 21.

Remdesivir is a new nucleotide analog in monophosphate structure and a pro-drug.[10] It is metabolized to the active form C-adenosine nucleoside triphosphate analog and inhibits viral RNA polymerase. Remdesivir is highly selective to the RNA polymerase of the virus, therefore, toxic side effects in humans are unlikely. Remdesivir has been shown to be effective against Ebola virus, Sars-CoV, MERS-CoV and its first clinical use was in the treatment of Ebola 22.

Molecular docking analysis results of antiviral drugs used in COVID-19 in the protein structure of Sars-Cov-2 Mpro (PDB ID: 6LU7) were compared on compounds used in other viral infections. Our study showed that bictegravir, remdevisir and lopinavir compounds in the Sars-Cov-2 Mpro structure showed high binding affinity compared to the antiviral compounds N3 inhibitor, according to our molecular docking results. However, the favipiravir, emtricitabine and lamuvidune compounds were detected very low binding affinity. Other antiviral compounds showed close binding affinity with the N3 inhibitor. Moreover, in the RMSD analysis performed, the accuracy of our docking results, those with RMSD values below 2 Å, indicate that the molecular docking study was successful.

Electrostatic complementarity between protein and ligand at the binding interface is vital for complex formation. Among the dominant types of electrostatic interactions are; hydrogen bonding, salt bridges and metal interactions. Hydrogen bonding is the most important directional interaction in biological macromolecules, which is known to impart stability to protein structure and selectivity to protein-ligand interactions 23,24. Generally, hydrogen bonding occurs between two electronegative atoms, one (donor) having a covalently bonded hydrogen atom and the other (acceptor) having a lone electron pair. The strong electrostatic attraction results from the attractive interaction between the partial positive charge on the hydrogen atom and the partial negative charge on the acceptor atom 25. Hydrophobic interactions are in contact with the nonpolar parts of the molecules thereby increasing entropy. Hydrophobic interactions are therefore entropy driven and have been shown to play a crucial role in ligand binding 26.

Recent studies of COVID-19 drug discovery have been reported by many studies on the idea of re-use of antiviral drugs. Researchers reported that re-uses of many existing antiviral drugs, such as remdesivir and favipiravir, have been officially approved for the treatment of COVID-19. Anti-HIV drugs such as ritonavir and nelfinavir have been proposed for re-use in COVID-19 by targeting Sars-Cov-2 Mpro 27. Antiviral drugs such as solutegravir, raltegravir, paritaprevir, bitegravir, and dolutegravir were also recommended in another report 28. Belhassan et al. in 2020, they described the optimal binding properties of oseltamivir derivatives with Sars-Cov-2 Mpro (Code PDB: 6LU7) and showed that oseltamivir derivatives bind well to the Sars Cov-2 Mpro construct^29^. Hagar et al. 2020, in their study, found that ribavirin showed good binding affinity in Sars-Cov-2 Mpro 30.

We could not find any study describing the structural binding properties of Emtricitabine, Entecavir, Lamivudine and Tenofovir antiviral compounds and Sars-Cov-2 Mpro structure. We have also explained the structural properties of these compounds.

## Conclusion

The Sars-Cov-2 epidemic, which has no proven treatment and vaccine yet, continues to affect the whole world. Many agents tried in the treatment are drugs that have been shown to be effective in previous epidemic experiences or are used with the expectation of being effective in vivo because they are effective in vitro. According to the results of molecular modeling, it has been determined that new agents can be used as an alternative to the antiviral agents used in coronavirus. It has been determined that antiviral agents with a docking score above -6.0 are suitable for use in clinical trials.
